# MMASleepNet: A multimodal attention network based on electrophysiological signals for automatic sleep staging

**DOI:** 10.3389/fnins.2022.973761

**Published:** 2022-08-16

**Authors:** Zheng Yubo, Luo Yingying, Zou Bing, Zhang Lin, Li Lei

**Affiliations:** School of Artificial Intelligence, University of Posts and Telecommunications, Beijing, China

**Keywords:** multimodal, attention network, automatic sleep staging, electrophysiological signals, features fusion

## Abstract

Pandemic-related sleep disorders affect human physical and mental health. The artificial intelligence (AI) based sleep staging with multimodal electrophysiological signals help people diagnose and treat sleep disorders. However, the existing AI-based methods could not capture more discriminative modalities and adaptively correlate these multimodal features. This paper introduces a multimodal attention network (MMASleepNet) to efficiently extract, perceive and fuse multimodal features of electrophysiological signals. The MMASleepNet has a multi-branch feature extraction (MBFE) module followed by an attention-based feature fusing (AFF) module. In the MBFE module, branches are designed to extract multimodal signals' temporal and spectral features. Each branch has two-stream convolutional networks with a unique kernel to perceive features of different time scales. The AFF module contains a modal-wise squeeze and excitation (SE) block to adjust the weights of modalities with more discriminative features and a Transformer encoder (TE) to generate attention matrices and extract the inter-dependencies among multimodal features. Our MMASleepNet outperforms state-of-the-art models in terms of different evaluation matrices on the datasets of Sleep-EDF and ISRUC-Sleep. The implementation code is available at: https://github.com/buptantEEG/MMASleepNet/.

## 1. Introduction

Sleep is an essential natural behavior for humans to maintain mental and physical health. Surveys show that ordinary people worldwide also have insomnia attributed to pandemic-related stress, anxiety, depression, and other mental health conditions during the new coronavirus pandemic (Semyachkina-Glushkovskaya et al., 2021). Survivors of COVID-19 are still bothered by insomnia (Taquet et al., 2021). The research found that adequate and effective sleep helps people improve the efficacy of COVID-19 vaccines (Benedict and Cedernaes, 2021), and sleeping in the rapid eye movement (REM) stage helps restore the brain's ability and remove waste from the brain (Van Alphen et al., 2021). Sleep staging helps ordinary people better understand their sleep quality and helps patients with insomnia or other related diseases to obtain better diagnoses and treatment (Pan et al., 2020).

Polysomnography (PSG) is the primary tool for assessing sleep in the laboratory and can be used for clinical and research purposes (Rundo and Downey, 2019). During polysomnography, EEG, EOG, EMG, and other electrophysiological signals are recorded as multimodal data and then used by professional doctors to divide sleep into distinct stages. The American Academy of Sleep Medicine (AASM) classifies each 30 s sleep epoch into five different stages (W, N1, N2, N3, and REM) (Chriskos et al., 2021). However, manual sleep staging requires professional knowledge and is highly time-consuming. Artificial intelligence technology helps to improve efficiency and has become a research hot spot of sleep staging in recent years.

There have been two main approaches widely adopted in sleep staging studies. Some researchers employed conventional machine learning methods, which mainly contained feature extraction algorithms and fed features into conventional classifiers (Awais et al., 2021). Due to the need for prior professional knowledge for feature extraction, these models have poor transfer ability, and non-end-to-end learning is significantly subject to subjective influence. For other researchers, deep learning methods were adopted due to their superior performance and less need for prior knowledge. Some studies designed convolutional neural networks (CNNs) for sleep staging (Supratak et al., 2017; Phan et al., 2018; Perslev et al., 2019; Jia et al., 2020). Some studies employed long short-term memory (LSTM) to capture the temporal context from the representative features in forward and backward directions (Supratak et al., 2017; Supratak and Guo, 2020; Neng et al., 2021). Recurrent Neural Networks (RNNS) were proposed to capture the temporal correlation of electrophysiological signals (Michielli et al., 2019). Attention mechanism and attention-based feature fusion have been widely used in multimodal representation learning (Huang et al., 2019, 2020; Lu et al., 2019; Wei et al., 2020; Zhang et al., 2020a,b,c; Desai and Johnson, 2021; Yu et al., 2021; Ma et al., 2022). The existing studies based on attention mechanisms usually used single-modal data such as EEG or EOG, which only focused on the inter-relationship among single modality features rather than cross-modal features (Eldele et al., 2021).

**Figure 1:**
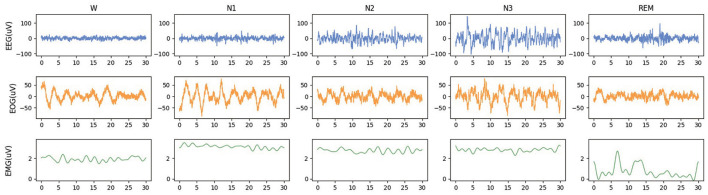
The waveforms of EEG, EOG, and EMG in each sleep stage. The data is randomly selected from the Sleep-EDF-78 dataset, and each epoch is 30 s.

The waveforms of EEG, EOG, and EMG in each sleep stage are shown in Figure 1. The signal characteristics of each modality among the five sleep stages are different, whether in the time domain or frequency domain. Observed from the time domain, signal amplitudes and cycles of different modalities signals are also various. Using EEG alone for sleep staging has been a feasible solution since EEG is the main basis of artificial sleep staging. It can also be observed that there are significant differences between the W stage and N1 stage in EOG waveforms, and the EMG waveforms are also helpful in identifying REM. Most studies chose EEG as the primary modality (Supratak et al., 2017). Some studies selected EOG signals which could be more convenient to acquire than EEG signals (Fan et al., 2021). Other studies also adopted EMG signals with more distinguishable features between the W and REM stages (Li et al., 2022). Further, it can be verified that the electrophysiological signals of the three modalities have complementary characteristics to sleep staging. By designing a neural network method of modality fusion, the accuracy of sleep staging can be improved. The existing multimodal sleep staging methods usually took EEG and EOG as the input of the model, and the fusion of multimodal features was mainly based on concatenation (Jia et al., 2020, 2021) without focusing on parts of the features.

**Figure 2:**
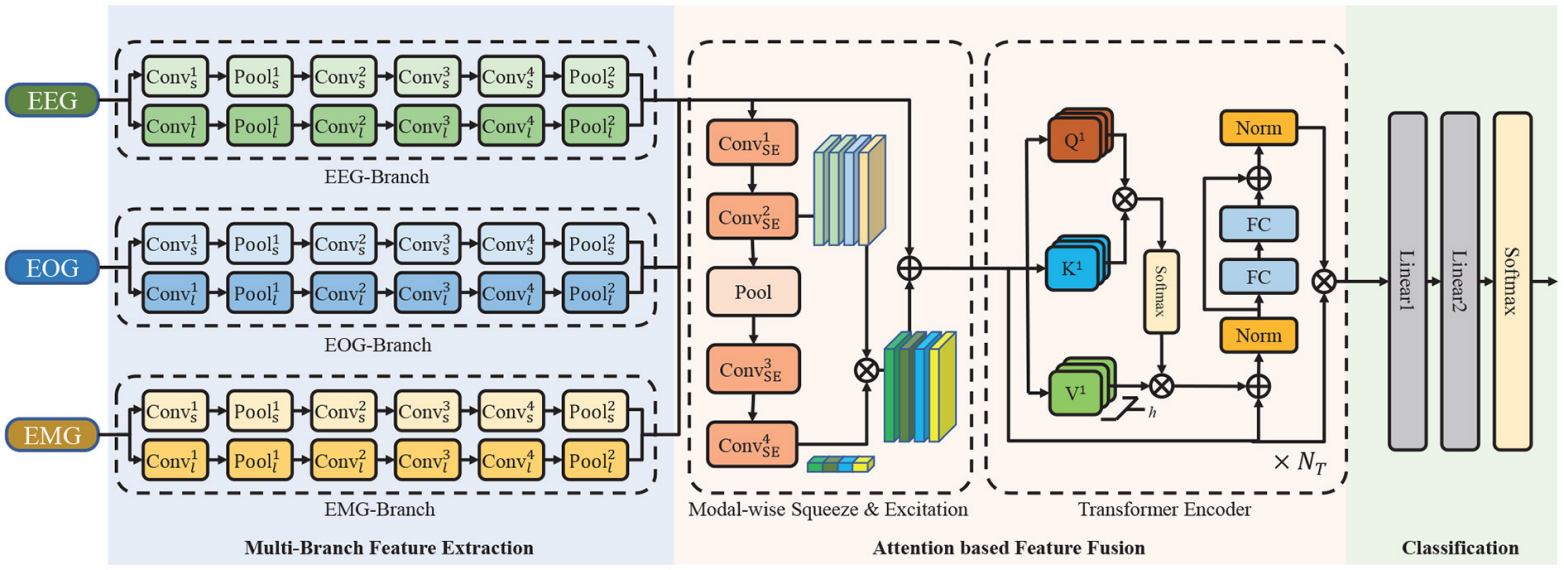
The architecture of the proposed network. It consists of a multi-branch feature extraction module, an attention based feature fusion module and a classification module. ⊕ is the point-wise addition and ⊗ is the point-wise multiplication. *Conv* is the convolutional layer, *Pool* is the pooling layer, *FC* is the fully connection layer, *Norm* is the normalization layer.

To efficiently extract multimodal features of EEG, EOG, and EMG, use the attention mechanism for feature fusion, and improve the accuracy of sleep staging, the multimodal attention network (MMASleepNet) is proposed, which has a multi-branch feature extraction module followed by an attention fusing module, as shown in Figure 2. The contributions of this paper are as follows.

1. The multi-branch feature extraction (MBFE) module is proposed, and unique kernels are specially designed based on the effective frequency band of three modalities.
2. The attention-based Feature Fusion (AFF) module is proposed, and modal-wise squeeze and excitation block are combined with Transformer Encoder to fuse the features of EEG, EOG, and EMG.
3. Experiments on four public datasets validate the effectiveness of the MMASleepNet. The results demonstrate that MMASleepNet outperforms all the baseline models in automatic sleep staging.

The context of this paper is as follows. Section 2 introduces data and methodology. The experiment design is described in the Section 3. Section 4 presents the results of experiments, and Section 5 analyses the results.

## 2. Materials and methods

### 2.1. Data description

Publicly available datasets were used for method evaluation, whose summary is shown in Table 1.

**Table 1:** Summary of the datasets and selected channels.

**Dataset**	**Subjects**	**Samples**	**W (%)**	**N1 (%)**	**N2 (%)**	**N3 (%)**	**REM (%)**	**Score method**	**k for k-fold**
Sleep-EDF-20	20	42,308	19.58	6.63	42.07	13.48	18.24	R&K	20
Sleep-EDF-78	78	195,479	33.74	11.01	35.37	6.67	13.22	R&K	10
ISRUC-Sleep-1	100	87,187	22.95	12.85	31.51	19.45	13.23	AASM	5
ISRUC-Sleep-3	10	8,589	20.44	14.04	30.12	22.90	12.50	AASM	10

#### 2.1.1. Sleep-EDF

The Sleep-EDF dataset contains two sub-datasets, namely, Sleep-EDF-20 and Sleep-EDF-78 (Goldberger et al., 2000). The Sleep-EDF-20 dataset contains 42308 epochs in 39 sleep cassette files collected from 20 subjects aged 25–34. The Sleep-EDF-78 dataset contains 195479 epochs in 153 sleep cassette files of 78 subjects aged 25–101. Each subject of the Sleep-EDF database contains 2 day-night PSG recordings except subjects 13, 36, and 52, whose one recording is lost due to device failure. The duration of each epoch is 30 s, and it has been labeled as {Wake, REM, N1, N2, N3, N4, MOVEMENT, UNKNOWN } by experts according to the R&K standard.

#### 2.1.2. ISRUC-sleep

ISRUC-Sleep-1 and ISRUC-Sleep-3 are the sub-datasets of the ISRUC-Sleep (Khalighi et al., 2016). The ISRUC-Sleep-1 dataset contains 69,671 epochs in 100 PSG data files collected from 100 subjects aged 20–85. The ISRUC-Sleep-3 dataset contains 8,589 epochs in 10 PSG data files collected from 10 subjects aged 30–58. Each recording contains 6 EEG channels (F3-A2, C3-A2, O1-A2, F4-A1, C4-A1, and O2-A1), 2 EOG channels (LOC-A2 and ROC-A1), 3 EMG channels (Chin EMG, left leg movements and right leg movements), and 1 ECG channel, and all signals were sampled at 200 Hz. The duration of each epoch is 30 s, and it has been labeled as {Wake, REM, N1, N2, N3 } by experts according to AASM standard.

For a fair comparison with baseline models, the following data preprocessing steps have been applied to the Sleep-EDF and ISRUC-Sleep datasets. The N3 and N4 are merged into N3 according to the AASM standard for the Sleep-EDF dataset. Then, MOVEMENT and UNKNOWN epochs are excluded. The signals of EEG (Fpz-Cz and Pz-Oz), EOG (ROC-LOC), and EMG (CHIN1-CHIN2) are adopted. For the ISRUC-Sleep dataset, the signals of EEG ( F3-A2, C3-A2, O1-A2, F3-A1, C4-A1, O2-A1), EOG (ROC-A1), and EMG (CHin-EMG) are adopted. For the four datasets, 30 min of wake epochs before and after sleep epochs are maintained to focus more on the sleep stages. In this study, all these signals are resampled at 100 Hz.

### 2.2. Method

Figure 2 illustrates the overall framework of MMASleepNet. The MMASleepNet consists of three main modules: multi-branch feature extraction (MBFE), attention-based feature fusion (AFF), and classification. The network can be trained and optimized using multimodal electrophysiological signals. Firstly, raw signals of each modality are processed into high-level features by the specially designed branches in the MBFE module. This module has several two-stream convolutional networks, which consist of a small kernel fully convolutional network (FCN) and a large kernel FCN to perceive features of different time scales. The AFF module includes a modal-wise squeeze and excitation (SE) block to adjust the weights of modalities with more discriminative features and TE layers to generate attention matrices and extract the inter-dependencies among multimodal features. Finally, the staging results can be obtained through the classification layer.

#### 2.2.1. Multi-branch feature extraction

In order to extract the features from the original multimodal data (EEG, EOG, and EMG), two-stream convolutional network branches are designed in the MBFE module. Each branch in the MBFE module consists of two FCN streams with four convolutional layers and two Max-Pooling layers. Referring to previous studies, the different sizes of convolutional kernels capture different scale features, making the feature matrix more comprehensive (Supratak et al., 2017). One FCN stream adopts a large kernel, and the other adopts a small kernel at the first convolutional layer. As the electrophysiological signals are sampled at 100 Hz, the convolutional layer with a kernel size of 500 extracts low-frequency information using 5-s windows. On the contrary, the small convolutional layer with a kernel size of 50 extracts the high-frequency information and detailed features using half-second windows. As the modalities have different interesting frequency ranges, the size of the convolutional kernel in the EEG branch is twice that of EOG and EMG. Due to EEG having higher classification accuracy in most cases, the number of convolutional kernels *d*_*EEG*_ for the EEG branch is also larger than *d*_*EOG*_ and *d*_*EMG*_ for EOG and EMG branches. The parameters of the MBFE module are given in Table 2. The leaky rectified linear unit (Leaky-ReLU) is employed as the activation function of each convolutional layer, which can be defined as follows:


(1) $$\begin{array}{r} LeakyReLU\left(x\right)=\{\begin{array}{cc} x, & x\geq 0\\ \alpha x, & x<0 \end{array} \end{array}$$

**Table 2:** Parameters of the MBFE module. *Size*_*k*_ is the size of convolutional kernel, *N* is the numbers of filters and *d*_*M*_ is the number of kernels at the last convolutional layer.

**Branch**	**Stream**	**Layers**						**d_*M*_**
		**Conv1D-1**	**MaxPooling-1**	**Conv1D-2**	**Conv1D-3**	**Conv1d-4**	**MaxPooling-2**	
EEG	Small	Size_*k*_ = 50 Stride = 8 *N* = 64	Size_*k*_ = 8 Stride = 8	Size_*k*_ = 8 Stride = 1 *N* = 128	Size_*k*_ = 8 Stride = 1 *N* = 128	Size_*k*_ = 8 Stride = 1 *N* = 128	Size_*k*_ = 4 Stride = 4	128
	Large	Size_*k*_ = 500 Stride = 64 *N* = 64	Size_*k*_ = 4 Stride = 4	Size_*k*_ = 6 Stride = 1 *N* = 128	Size_*k*_ = 6 Stride = 1 *N* = 128	Size_*k*_ = 6 Stride = 1 *N* = 128	Size_*k*_ = 2 Stride = 2	128
EOG	Small	Size_*k*_ = 25 Stride = 8 *N* = 32	Size_*k*_ = 8 Stride = 8	Size_*k*_ = 8 Stride = 1 *N* = 64	Size_*k*_ = 8 Stride = 1 *N* = 64	Size_*k*_ = 8 Stride = 1 *N* = 64	Size_*k*_ = 4 Stride = 4	64
	Large	Size_*k*_ = 250 Stride = 64 *N* = 32	Size_*k*_ = 4 Stride = 4	Size_*k*_ = 6 Stride = 1 *N* = 64	Size_*k*_ = 6 Stride = 1 *N* = 64	Size_*k*_ = 6 Stride = 1 *N* = 64	Size_*k*_ = 2 Stride = 2	64
EMG	Small	Size_*k*_ = 25 Stride = 8 *N* = 32	Size_*k*_ = 8 Stride = 8	Size_*k*_ = 8 Stride = 1 *N* = 64	Size_*k*_ = 8 Stride = 1 *N* = 64	Size_*k*_ = 8 Stride = 1 *N* = 64	Size_*k*_ = 4 Stride = 4	64
	Large	Size_*k*_ = 250 Stride = 64 *N* = 32	Size_*k*_ = 4 Stride = 4	Size_*k*_ = 6 Stride = 1 *N* = 64	Size_*k*_ = 6 Stride = 1 *N* = 64	Size_*k*_ = 6 Stride = 1 *N* = 64	Size_*k*_ = 2 Stride = 2	64

The Leaky-ReLU can solve the zero gradient vanishing problems for negative values, which are essential for the following modules. Dropout layers are applied after the first Max-Pooling in both streams and after the concatenation of both streams to reduce overfitting. The input $X_{M}\in {\mathbb{R}}^{3\times C_{M}\times N}$ are fed into the MBFE module for extracting the multimodal features where *M* ∈ {*EEG, EOG, EMG*} represent modalities, and *C*_*M*_ is the number of channels for modal *M*, *N* = *SampleRate* × *EpochTime* is the samples for a single channel in one epoch. The operation is formalized as follows:


(2) $$\begin{array}{r} F_{M}=FCN_{M}^{s}\left(X_{M}\right)\|FCN_{M}^{l}\left(X_{M}\right)\;\in {\mathbb{R}}^{d_{M}\times l} \end{array}$$

where *FCN*^*s*^ and *FCN*^*l*^ represent FCN stream with the small and large convolutional kernel and || is the concatenate operation. $F_{EEG}\in {\mathbb{R}}^{d_{EEG}\times l}$ is divided into $F_{EEG}^{i}\in {\mathbb{R}}^{d\times l}$, where $d=\frac{1}{2}d_{EEG},i\in \left\{1,2\right\}$ to align with the *F*_*EOG*_ and *F*_*EMG*_ for the following concatenate operation. A new dimension is created on each modalities' features, and the concatenate operation is formalized as follows:


(3) $$\begin{array}{r} F=\left[F_{EEG}^{1},F_{EEG}^{2},F_{EOG},F_{EMG}\right]\;\in {\mathbb{R}}^{4\times d\times l} \end{array}$$

where [·] is the concatenate operation on the newly created modal dimension. A feature map *F* that contains different modalities of information is obtained through above operations.

#### 2.2.2. Attention-based feature fusion

The AFF module is designed for fusing features extracted by the MBFE module. The architecture shown in Figure 2 is designed based on attention methods. AFF module consists of a modal-wise SE block and TE layers.

##### 2.2.2.1. Modal-wise SE

The modal-wise SE block is proposed based on the SENet (Hu et al., 2020). Different from the SENet using 1D convolutional and Max-Pooling layers, as shown in Figure 2, 2D convolutional and Max-Pooling layers are implemented to reconstruct the input features. Given a feature map *F* ∈ ℝ^4 × *d* × *l*^, two convolution operations are applied to *F* such that $F^{\prime }=Conv_{2}\left(Conv_{1}\left(F\right)\right)$ and *F*′ has the exact dimensions as the input feature map. Global Average Pooling is performed along the spatial dimensions, and *F*′ is turned into *S* = {*S*_1_, *S*_2_, *S*_3_, *S*_4_}. Two additional 2D convolutional layers replace the full connection layers in SENet to reconstruct *S* further. The first layer followed with ReLU activation function designed to reduce the dimensions of *F*, and the second layer followed with Sigmoid layer aims to increase the dimensions. The operation is formalized as follows:


(4) $$\begin{array}{r} E=\text{Sigmoid}\left(\text{Con}{\text{v}}_{2}\left(\text{ReLU}\left(\text{Con}{\text{v}}_{1}\left(S\right)\right)\right)\right)\;\in {\mathbb{R}}^{4\times d\times l} \end{array}$$

where *Conv*_1_ and *Conv*_2_ are the 2D convolution operations, sigmoid and ReLU are the activation functions and ReLU(*x*) = max(0, *x*). The output dimension matches the number of input modalities. It characterizes the global distribution of responses over features. Then, the feature map *F* is scaled by *E*:


(5) $$\begin{array}{r} O_{SE}=F⊕\left(F⊗E\right)\;\in {\mathbb{R}}^{4\times d\times l} \end{array}$$

where ⊕ is the point-wise addition and ⊗ is the point-wise multiplication, *O*_*SE*_ is the output of the modal-wise SE block. Modal-wise SE block adaptively learns the correlation among multiple modalities and the attention of different modalities.

##### 2.2.2.2. Transformer encoder

As shown in Figure 1, each TE layer comprises two core modules: multi-head attention and position-wise feed-forward network. Multi-head attention consists of *H* attention modules. Firstly, *H* different linear projections are applied to the input, and the result is mapped to parallel queries, keys, and values. Secondly, dot-product is performed on *Q*_*i*_ and *K*_*i*_ to calculate a similarity score. A normalization operation is applied to stabilize the gradient. Then, the Softmax operation calculates the weight for *V*_*i*_, and another dot-product is applied. Finally, all the *A*_*i*_ are concatenated together to produce the final output. The operations can be formulated as follows:


(6) $$\begin{array}{r} Q_{i}=ZW_{i}^{Q},K_{i}=ZW_{i}^{K},V_{i}=ZW_{i}^{V},0<i\leq H \end{array}$$

(7) $$\begin{array}{r} A_{i}=\text{Softmax}\left(\frac{Q_{i}\cdot K_{i}^{\text{T}}}{\sqrt{d}}\right)\cdot V_{i} \end{array}$$

(8) $$\begin{array}{r} MA=A_{1}\|A_{2}\|...\|A_{H} \end{array}$$

where *Z* ∈ ℝ^4*l* × *d*^ is the input of the TE layer. $W_{i}^{Q}$, $W_{i}^{K}$, $W_{i}^{V}$ $\in {\mathbb{R}}^{d\times \frac{d}{H}}$ are learnable weights of linear projections, *d* is the column length of *Z*, and || is the concatenate operation. Residual layers are applied as Equation 9. The position-wise feed-forward network consists of two linear transformations with ReLU activation as follows:


(9) $$\begin{array}{r} O^{1}=\text{LayerNorm}\left(MA+Z\right) \end{array}$$

(10) $$\begin{array}{r} O^{2}=\text{ReLU}\left(O^{1}W_{1}+b_{1}\right)W_{2}+b_{2} \end{array}$$

where $W_{1}\in {\mathbb{R}}^{d\times d_{FF}}$, $W_{2}\in {\mathbb{R}}^{d_{FF}\times d}$ are learnable weight matrices $b_{1}\in {\mathbb{R}}^{d_{FF}}$, $b_{2}\in {\mathbb{R}}^{d}$ is learnable biases. *d*_*FF*_ is the middle dimension of the feed-forward network. Then the output of the attention-based feature fusion module *O*_*AF*_ can be obtained as follow:


(11) $$\begin{array}{r} O_{TE}=\text{LayerNorm}\left(O^{1}⊕O^{2}\right) \end{array}$$

(12) $$\begin{array}{r} O_{AF}=Z⊗O_{TE} \end{array}$$

where *Z* is the flattened output of modal-wise SE block, and *O*_*TE*_ is the output of the TE layer. Then the *O*_*AF*_ is fed into two linear layers for the final classification.

## 3. Experiment

### 3.1. Baseline methods

Our method has been compared with the three baseline models: AttnSleepNet, SleepPrintNet, and SalientSleepNet. The publicly available codes have been used for AttnSleepNet, whereas SleepPrintNet and SalientSleepNet were re-implemented. For a fair comparison, all models were trained and tested on the same data partition with the same random seeds. Brief descriptions for models are as follows:

- **AttnSleepNet (Eldele et al.**, **2021****):** AttnSleepNet deploys a custom CNN architecture followed by a multi-head attention mechanism and causal convolutions.
- **SleepPrintNet (Jia et al.**, **2020****):** An EEG temporal feature extraction module, an EEG spectral-spatial feature extraction module, and two multimodal feature extraction modules are combined and classified.
- **SalientSleepNet (Jia et al.**, **2021****):** A fully convolutional network based on the *U*^2^-Net architecture. Two independent *U*^2^-like streams are composed to extract the features from multimodal data.

### 3.2. Experiment settings

To evaluate the performance of models, subjects in each dataset were divided into several groups using k-fold cross-validation. For each fold, one group of subjects was selected as validation data. The remaining k-1 groups were selected as training data. Finally, four performance matrices were calculated by combining the predicted sleep stages of all *k* test groups. For the MMASleepNet, the Adam optimizer with the learning rate of 1e-4 was applied. The weight decay of Adam was set to 1e-3, the betas (b1, b2) were used as (0.9, 0.999), respectively, and the epsilon value was 1e-08. The parameters of the MBFE module are introduced in Table 1. The TE block has only one encoder layer with four heads. The training epoch is 150. Weighted cross-entropy loss was adopted as follows:


(13) $$\begin{array}{r} L=-\frac{1}{N}\overset{N}{\underset{i=1}{\sum }}\overset{C}{\underset{c=1}{\sum }}\omega_{c}y_{i}^{c}\text{log}\left(p_{i}^{c}\right) \end{array}$$

where *N* is the batch size, *C* is the number of classes, $y_{i}^{c}$ is the true label, and $p_{i}^{c}$ is the predicted label of *i*-th samples for class *c*. ω_*c*_ ∈ {1.0, 1.80, 1.0, 1.25, 1.20} is the weight of class *c*.

For a fair comparison, all baseline models and proposed methods used the same dataset partitioning during training and evaluation. A number of experiments were conducted to find the best hyperparameters of the proposed MMASleepNet. The hyper-parameters of baseline models were set as introduced best in their article or open source codes. The train and validation codes are available at https://github.com/buptantEEG/MMASleepNet/.

### 3.3. Evaluation matrices

Four matrices were adopted to evaluate the performance of sleep staging models, namely, accuracy (ACC), macro-averaged F1-score (MF1), Cohen Kappa (κ), and the macro-averaged G-mean (MGm). Given True Positives (*TP*_*i*_), False Positives (*FP*_*i*_), True Negatives (*TN*_*i*_), and False Negatives (*FN*_*i*_) for the *i*-th class, the overall accuracy of ACC, MF1, κ, and MGm are defined as follows:


(14) $$\begin{array}{r} ACC=\frac{\sum_{c=1}^{C}TP_{c}}{N} \end{array}$$

(15) $$\begin{array}{r} \kappa =\frac{ACC-p_{e}}{1-p_{e}} \end{array}$$

(16) $$\begin{array}{r} MF1=\frac{1}{C}\overset{C}{\underset{c=1}{\sum }}\frac{2\times Precision_{c}\times Recall_{c}}{Precision_{c}+Recall_{c}} \end{array}$$

(17) $$\begin{array}{r} MGm=\frac{1}{C}\overset{C}{\underset{c=1}{\sum }}\sqrt{Specificity_{c}\times Recall_{C}} \end{array}$$

where $p_{e}=\frac{\overset{C}{\underset{c=1}{\sum }}a_{c}\times b_{c}}{N\times N}$, $Precision_{c}=\frac{TP_{c}}{TP_{c}+FP_{c}}$, $Recall_{c}=\frac{TP_{c}}{TP_{c}+FN_{c}}$ and $Specificity_{c}=\frac{TN_{c}}{TN_{c}+FP_{c}}$, *a*_*c*_ is the number of samples of class *c*, *b*_*c*_ is the number of samples predicted as the class *c*. *C* is the number of classes, and *N* is the total number of samples.

## 4. Results

### 4.1. Results comparison with baselines

Table 3 shows the comparison among AttnSleepNet, SleepPrintNet, SalientSleepNet, and our MMASleepNet. The single-modal method AttnSleepNet obtained the lowest accuracy of the four models. The multimodal approaches, SleepPrintNet and SalientSleepNet, achieve higher accuracy than the single-modal method. The multimodal model can capture different electrophysiological signal features diversity compared to single-modal signals. In addition, the accuracy of the proposed MMASleepNet reaches 87.30, 82.67, 79.02, and 81.92%, which is higher than all the baseline models. The MF1, κ, and MGm of MMASleepNet outperform all baseline models on the four datasets, which means that the MMASleepNet is better at adapting to unbalanced data and should get better accuracy when the classes are balanced.

**Table 3:** Comparison among MMASleepNet and baseline models.

**Dataset**	**Method**	**Per-class F1-score**					**Overall matrices**			

		W	N1	N2	N3	REM	*ACC*	*MF*1	κ	*MGm*
Sleep-EDF-20	AttnSleepNet	79.02	32.70	87.03	85.67	72.36	79.10	71.35	71.43	66.34
	SleepPrintNet	88.77	47.99	86.72	86.21	80.26	83.08	77.99	76.67	76.34
	SalientSleepNet	90.79	49.86	89.03	84.77	**88.44**	86.28	80.58	81.02	77.32
	MMASleepNet	**92.20**	**54.75**	**89.70**	**90.20**	86.41	**87.30**	**82.65**	**82.63**	**81.67**
Sleep-EDF-78	AttnSleepNet	92.08	36.98	84.70	**81.63**	73.61	81.12	73.80	73.75	68.64
	SleepPtintNet	92.65	47.39	83.59	79.97	78.75	81.64	76.47	74.70	74.27
	SalientSleepNet	92.28	**50.52**	84.37	71.17	**84.19**	82.61	76.51	75.92	73.42
	MMASleepNet	**92.85**	49.05	**84.94**	81.26	79.75	**82.67**	**77.60**	**76.12**	**76.06**
ISRUC-SLEEP-1	AttnSleepNet	84.19	43.80	71.52	81.93	61.12	71.65	68.53	63.70	67.43
	SleepPtintNet	79.12	40.12	58.22	68.80	73.67	65.40	63.99	56.02	62.47
	SalientSleepNet	85.24	51.34	76.41	83.50	79.25	76.95	75.15	70.31	74.25
	MMASleepNet	**87.83**	**54.03**	**77.05**	**85.29**	**83.31**	**79.02**	**77.51**	**73.02**	**76.79**
ISRUC-SLEEP-3	AttnSleepNet	67.58	26.91	66.31	84.08	54.33	64.24	59.85	54.88	55.83
	SleepPrintNet	85.15	52.53	74.95	87.28	74.84	76.88	74.95	70.29	73.69
	SalientSleepNet	78.37	50.64	77.33	**87.99**	75.47	76.11	73.96	69.39	73.20
	MMASleepNet	**88.87**	**59.57**	**82.00**	87.00	**86.87**	**81.92**	**80.64**	**76.79**	**80.00**

*The best values on each dataset are highlighted in bold*.

**Figure 3:**
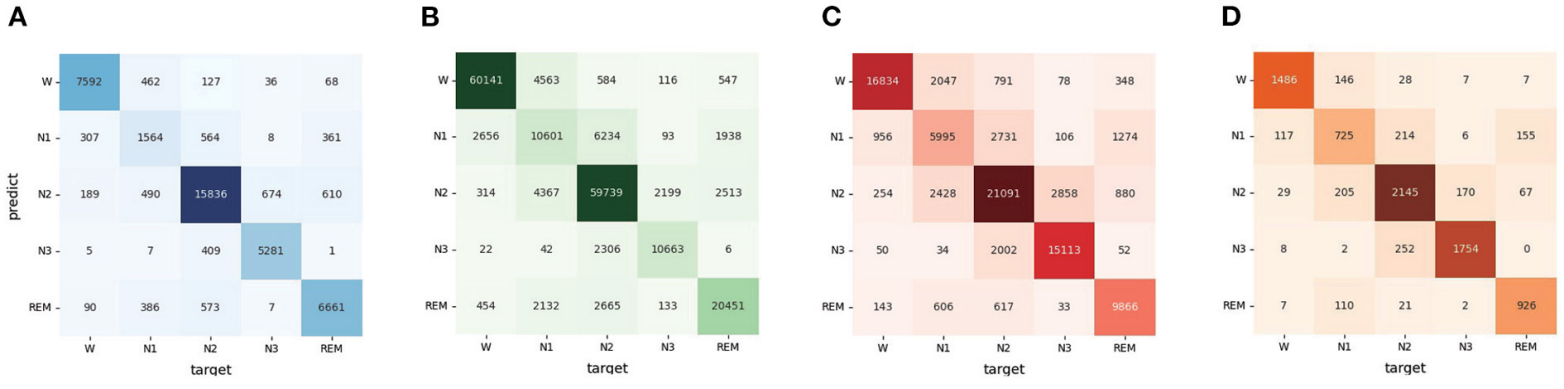
The confusion matrices of MMASleepNet, **(A)** is the confusion matrix valuated on SleepEDF-20 dataset, **(B)** is the confusion matrix valuated on SleepEDF-78 dataset, **(C)** is the confusion matrix valuated on ISRUC-Sleep-1 dataset, **(D)** is the confusion matrix valuated on ISRUC-Sleep-3 dataset.

According to the confusion matrix in Figure 3, the classification accuracy of W, N2, N3, and REM is relatively high both on the Sleep-EDF dataset and ISRUC dataset. The accuracy of recognizing stage N1 is lower than in other stages, which is related to the insufficient N1 samples in the sleep records. Table 3 shows that the MMASleepNet obtained a higher F1 score for stage N1 on the smaller datasets Sleep-EDF-20, ISRUC-Sleep-1, and ISRUC-Sleep-3, indicating that the MMASleepNet performs better than the baseline methods for imbalanced categories. The results demonstrate the advantages of MMASleepNet in automatic sleep staging with the proposed feature extracting and fusion operations applied to multimodal electrophysiological signals.

### 4.2. Ablation experiments

The MMASleepNet consists of an MBFE module, modal-wise SE block, and Transformer Encoder layers. To analyze the influence of each module and to prove the effectiveness of each modality used in MMASleepNet, the ablation experiment was designed on the Sleep-EDF-20 dataset as follows:

- **MBFE(basic):** This model is only MBFE module input with EEG, EOG, and EMG signals. The features obtained from MBFE are fed into a linear classification module for sleep staging.
- **MBFE+TE:** This model adds TE layers based on the basic model input with EEG, EOG, and EMG signals.
- **MMASleepNet1:** The completely MMASleepNet with MBFE, modal-wise SE block, and TE layers, only input with EEG signals.
- **MMASleepNet2:** MMASleepNet input with EEG and EOG signals.
- **MMASleepNet3:** MMASleepNet input with EEG, EOG, and EMG signals.

**Figure 4:**
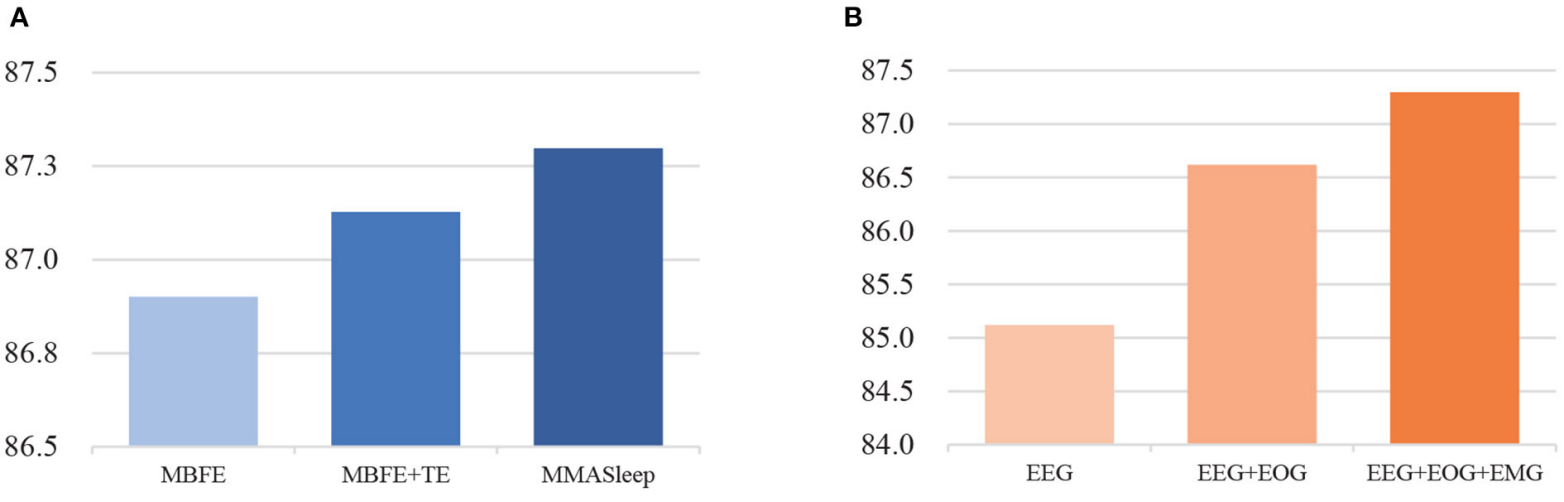
The results of ablation experiments, panel **(A)** is for the module ablation, panel **(B)** is for the modalities ablation.

Figure 4 presents the results of ablation experiments. Figure 4A shows that the attention-based feature fusion module improves the performance of the basic model. The modal-wise SE block helps the MMASleepNet achieve higher accuracy than only using TE layers. Figure 4B shows that MMASleepNet input with more modalities achieves higher accuracy. The model training with EOG and EMG performed better than with EEG alone.

## 5. Discussion

This study proposes a multimodal attention network for sleep staging using EEG, EOG, and EMG. The basis of using EEG, EOG, and EMG for sleep staging is that the PSG data collected in sleep health monitoring commonly includes multimodal electrophysiological signals. According to the experimental results, there are complementary features related to sleep stages among multiple modalities. The result shows that the proposed MMASleepNet achieves the highest classification performance on four publicly available datasets. Compared with the single-modality model AttnSleepNet, the proposed MMASleepNet can be fed with more data of multiple modalities, which means more information to extract and leads to big improvements in four evaluation matrices. Compared with the multimodal methods SleepPrintNet and SalinetSleepNet, MMASleepNet contains better-designed feature extraction methods and feature fusion methods for multimodal electrophysiological signals. The modal-wise SE block construct fusion of features adopted 2D convolutional, which makes it reasonable for complementary modalities. The SalientSleepNet also achieves high accuracy, but the high complexity of the modal led to lower training speed. The number of MMASleepNet parameters is 1.5M. The MMASleepNet has lower computation complexity and floating-point operations, improving the training speed. The AttnSleepNet, SleepPrintNet, SalientSleepNet, and the proposed MMASleepNet cost 0.4, 0.9, 7, and 1 h for 100 training epochs on the NVIDIA GeForce RTX 2080 Ti, respectively. Considering the accuracy and the training speed, the MMASleepNet performs better.

The ablation experiment results verify each module's effectiveness in the proposed MMASleepNet for automatic sleep staging. The ablation experiments in the first step verified that MMASleepNet fed with the data of three modalities achieves better results than a single modality. This preliminary verifies that the data of different modalities correlate with sleep stages and can be combined to obtain more time-frequency information. Features extracted from EOG and EMG complement those extracted from EEG only.

**Figure 5:**
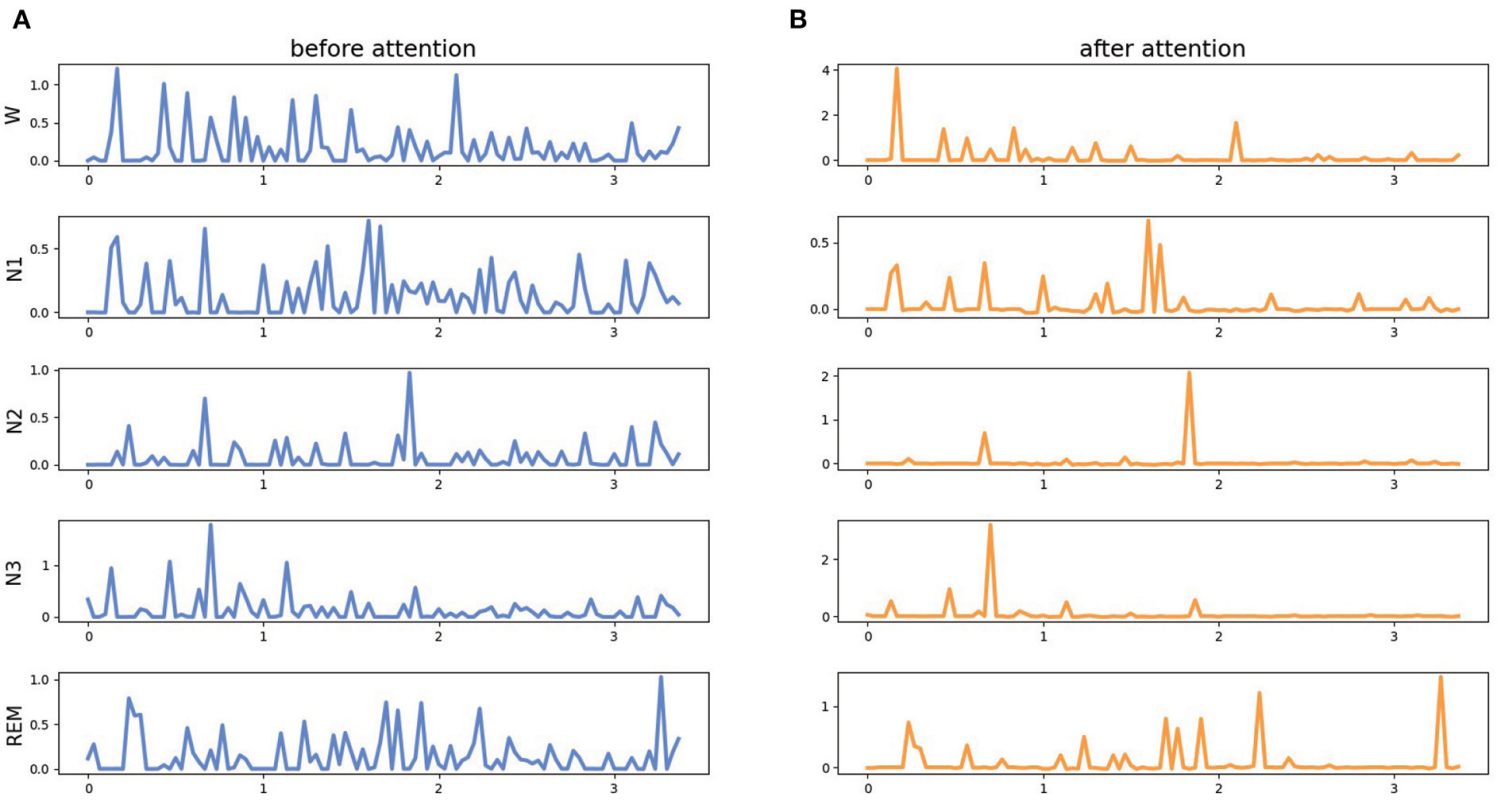
The features before and after attention mechanism of MMASleepNet. The data was selected randomly from the Sleep-EDF-20 dataset. Panel **(A)** is before the attention module, panel **(B)** is for the modalities ablation.

Figure 5 shows the down-sampled features before and after the AFF module. The main difference is that the features become more focused after the AFF module. The features after attention are easier to be distinguished using the same classifier, and the classifier is easier to converge. The visualized features show that the separability of the fused multimodal features can be enhanced with the attention mechanism, and the neural network observes more detailed differences.

Unlike previous studies, the proposed MMASleepNet has a more effective feature fusion module, especially the modal-wise SE block, rather than a simple concatenate operation on different modalities' features. Although the model complexity has increased slightly, the model understands the relationship among different modalities. MMASleepNet can extract effective information from different modalities and fully use multimodal information by fusing the features with attention methods. Experiment results show that MMASleepNet achieves state-of-the-art performance. A series of ablation experiments have shown that different modules of the model contribute to the sleep staging task. MMASleepNet improves the accuracy of sleep staging, which provides a solution for multimodal sleep monitoring and is helpful for people to understand sleep status and improve their sleep quality.

Sleep disturbances increased significantly during the pandemic (Semyachkina-Glushkovskaya et al., 2021). For studying whether there has been a change in sleep disturbances, new sleep data set during the pandemic and new methods need to be supported, and deeper analysis based on statistical principles is required. Sleep staging is a fundamental application that helps study sleep disturbances during a real pandemic. With the existing standards, the definition of sleep stages will not be easily changed. Data set during the pandemic will be collected, and new methods for deeper analysis will be devised to raise awareness of the pandemic. The interpretability of the model and transfer learning method will be investigated to improve generalization across subjects and datasets.

## Data availability statement

The original contributions presented in the study are included in the article/supplementary material, further inquiries can be directed to the corresponding author. Publicly available datasets were analyzed in this study. The Sleep-EDF can be found in https://www.physionet.org/content/sleep-edfx/1.0.0/ and the ISRUC-Sleep can be found in https://sleeptight.isr.uc.pt/.

## Author contributions

ZY, LY, and ZB contributed to the conception of the study. ZY and LY realized proposed model and baseline models, performed the experiment, wrote the manuscript. ZY contributed significantly to analysis. LY and ZB contributed to the data curation, data analyses, and manuscript preparation. ZB helped to perform the analysis with constructive discussions. ZL and LL contributed to the funding acquisition and supervision. All authors contributed to the article and approved the submitted version.

## Funding

This work was supported by the National Natural Science Foundation of China (Grant No. 62176024) and project A02B01C01-201916D2.

## Conflict of interest

The authors declare that the research was conducted in the absence of any commercial or financial relationships that could be construed as a potential conflict of interest.

## Publisher's note

All claims expressed in this article are solely those of the authors and do not necessarily represent those of their affiliated organizations, or those of the publisher, the editors and the reviewers. Any product that may be evaluated in this article, or claim that may be made by its manufacturer, is not guaranteed or endorsed by the publisher.

## References

[B1] Awais M. Long X. Yin B. Abbasi S. F. Akbarzadeh S. Lu C.. (2021). A hybrid DCNN-SVM model for classifying neonatal sleep and wake states based on facial expressions in video. IEEE J. Biomed. Health Inform. 25, 1441–1449. 10.1109/JBHI.2021.3073632 33857007

[B2] Benedict C. Cedernaes J. (2021). Could a good night's sleep improve COVID-19 vaccine efficacy? Lancet Respirat. Med. 9, 447–448. 10.1016/S2213-2600(21)00126-0 33721558 PMC7954467

[B3] Chriskos P. Frantzidis C. A. Nday C. M. Gkivogkli P. T. Bamidis P. D. Kourtidou-Papadeli C. (2021). A review on current trends in automatic sleep staging through bio-signal recordings and future challenges. Sleep Med. Rev. 55, 101377. 10.1016/j.smrv.2020.101377 33017770

[B4] Desai K. Johnson J. (2021). Virtex: learning visual representations from textual annotations, in Proceedings of the IEEE/CVF Conference on Computer Vision and Pattern Recognition (CVPR), 11162–11173. 10.1109/CVPR46437.2021.01101

[B5] Eldele E. Chen Z. Liu C. Wu M. Kwoh C.-K. Li X.. (2021). An attention-based deep learning approach for sleep stage classification with single-channel EEG. IEEE Trans. Neural Syst. Rehabil. Eng. 29, 809–818. 10.1109/TNSRE.2021.3076234 33909566

[B6] Fan J. Sun C. Long M. Chen C. Chen W. (2021). EOGNet: a novel deep learning model for sleep stage classification based on single-channel EOG signal. Front. Neurosci. 15, 573194. 10.3389/fnins.2021.573194 34321991 PMC8311494

[B7] Goldberger A. L. Amaral L. A. N. Glass L. Hausdorff J. M. Ivanov P. C. Mark R. G.. (2000). PhysioBank, PhysioToolkit, and PhysioNet: components of a new research resource for complex physiologic signals. Circulation. 101, e215–e220. 10.1161/01.cir.101.23.e215 10851218

[B8] Hu J. Shen L. Albanie S. Sun G. Wu E. (2020). Squeeze-and-excitation networks. IEEE Trans. Pattern Anal. Mach. Intell. 42, 2011–2023. 10.1109/TPAMI.2019.2913372 31034408

[B9] Huang Z. Xu X. Ni J. Zhu H. Wang C. (2019). Multimodal representation learning for recommendation in internet of things. IEEE Internet Things J. 6, 10675–10685. 10.1109/JIOT.2019.2940709

[B10] Huang Z. Xu X. Zhu H. Zhou M. (2020). An efficient group recommendation model with multiattention-based neural networks. IEEE Trans. Neural Netw. Learn. Syst. 31, 4461–4474. 10.1109/TNNLS.2019.2955567 31944999

[B11] Jia Z. Lin Y. Wang J. Wang X. Xie P. Zhang Y. (2021). SalientSleepNet: Multimodal salient wave detection network for sleep staging, in Proceedings of the Thirtieth International Joint Conference on Artificial Intelligence, ed Zhou Z. -H. (International Joint Conferences on Artificial Intelligence Organization), 2614–2620. 10.24963/ijcai.2021/360

[B12] Jia Z. Cai X. Zheng G. Wang J. Lin Y. (2020). SleepPrintNet: a multivariate multimodal neural network based on physiological time-series for automatic sleep staging. IEEE Trans. Artif. Intell. 1, 248–257. 10.1109/TAI.2021.3060350

[B13] Khalighi S. Sousa T. Santos J. M. Nunes U. (2016). ISRUC-Sleep: A comprehensive public dataset for sleep researchers. Comput. Methods Prog. Biomed. 124, 180–192. 10.1016/j.cmpb.2015.10.013 26589468

[B14] Li Y. Xu Z. Zhang Y. Cao Z. Chen H. (2022). Automatic sleep stage classification based on two-channel EOG and one-channel EMG. Physiol. Meas. 43. 10.1088/1361-6579/ac6bdb 35487205

[B15] Lu J. Batra D. Parikh D. Lee S. (2019). Vilbert: Pretraining task-agnostic visiolinguistic representations for vision-and-language tasks. Advances in neural information processing systems 32.

[B16] Ma J. Tang L. Fan F. Huang J. Mei X. Ma Y. (2022). SwinFusion: cross-domain long-range learning for general image fusion via swin transformer. IEEE/CAA J. Automat. Sin. 9, 1200–1217. 10.1109/JAS.2022.105686

[B17] Michielli N. Acharya U. R. Molinari F. (2019). Cascaded LSTM recurrent neural network for automated sleep stage classification using single-channel EEG signals. Comput. Biol. Med. 106, 71–81. 10.1016/j.compbiomed.2019.01.013 30685634

[B18] Neng W. Lu J. Xu L. (2021). CCRRSleepNet: a hybrid relational inductive biases network for automatic sleep stage classification on raw single-channel EEG. Brain Sci. 11, 456. 10.3390/brainsci11040456 33918506 PMC8065855

[B19] Pan Q. Brulin D. Campo E. (2020). Current status and future challenges of sleep monitoring systems: systematic review. JMIR Biomed. Eng. 5, e20921. 10.2196/2092133351435

[B20] Perslev M. Jensen M. Darkner S. Jennum P. J. Igel C. (2019). U-Time: a fully convolutional network for time series segmentation applied to sleep staging, in Advances in Neural Information Processing Systems, Vol. 32, eds Wallach H. Larochelle H. Beygelzimer A. d'Alché-Buc F. Fox E. Garnett R. (Red Hook, NY: Curran Associates, Inc.), 4415-4426.

[B21] Phan H. Andreotti F. Cooray N. Chén O. Y. De Vos M. (2018). Joint classification and prediction CNN framework for automatic sleep stage classification. IEEE Trans. Biomed. Eng. 66, 1285–1296. 10.1109/TBME.2018.2872652 30346277 PMC6487915

[B22] Rundo J. V. Downey R. (2019). Chapter 25: Polysomnography, in Clinical Neurophysiology: Basis and Technical Aspects, volume 160 of Handbook of Clinical Neurology, eds Levin K. H. Chauvel P. (Amsterdam: Elsevier), 381–392.

[B23] Semyachkina-Glushkovskaya O. Mamedova A. Vinnik V. Klimova M. Saranceva E. Ageev V.. (2021). Brain mechanisms of COVID-19-sleep disorders. Int. J. Mol. Sci. 22:6917. 10.3390/ijms22136917 34203143 PMC8268116

[B24] Supratak A. Dong H. Wu C. Guo Y. (2017). DeepSleepNet: a model for automatic sleep stage scoring based on raw single-channel EEG. IEEE Trans. Neural Syst. Rehabil. Eng. 25, 1998–2008. 10.1109/TNSRE.2017.2721116 28678710

[B25] Supratak A. Guo Y. (2020). TinySleepNet: an efficient deep learning model for sleep stage scoring based on raw single-channel EEG, in 2020 42nd Annual International Conference of the IEEE Engineering in Medicine Biology Society (EMBC), 641–644. 10.1109/EMBC44109.2020.9176741 33018069

[B26] Taquet M. Geddes J. R. Husain M. Luciano S. Harrison P. J. (2021). 6-month neurological and psychiatric outcomes in 236 379 survivors of COVID-19: a retrospective cohort study using electronic health records. Lancet Psychiatry 8, 416–427. 10.1016/S2215-0366(21)00084-5 33836148 PMC8023694

[B27] Van Alphen B. Semenza E. R. Yap M. Van Swinderen B. Allada R. (2021). A deep sleep stage in drosophila with a functional role in waste clearance. Sci. Adv. 7, eabc2999. 10.1126/sciadv.abc2999 PMC7817094 33523916

[B28] Wei X. Zhang T. Li Y. Zhang Y. Wu F. (2020). Multi-modality cross attention network for image and sentence matching, in 2020 IEEE/CVF Conference on Computer Vision and Pattern Recognition (CVPR), 10938–10947. 10.1109/CVPR42600.2020.01095

[B29] Yu F. Tang J. Yin W. Sun Y. Tian H. Wu H.. (2021). ERNIE-ViL: knowledge enhanced vision-language representations through scene graphs, in Proceedings of the AAAI Conference on Artificial Intelligence, 3208–3216.

[B30] Zhang C. Yang Z. He X. Deng L. (2020a). Multimodal intelligence: representation learning, information fusion, and applications. IEEE J. Select. Top. Signal Process. 14, 478–493. 10.1109/JSTSP.2020.2987728

[B31] Zhang K. Su Y. Guo X. Qi L. Zhao Z. (2020b). MU-GAN: facial attribute editing based on multi-attention mechanism. IEEE/CAA J. Automat. Sin. 8, 1614–1626. 10.1109/JAS.2020.1003390

[B32] Zhang Y. Xu B. Zhao T. (2020c). Convolutional multi-head self-attention on memory for aspect sentiment classification. IEEE/CAA J. Automat. Sin. 7, 1038–1044. 10.1109/JAS.2020.1003243

